# Assessment of the availability of repurposed orphan drugs in India

**DOI:** 10.1371/journal.pgph.0001498

**Published:** 2023-09-27

**Authors:** Khujith Rajueni, Mohua Chakraborty Choudhury

**Affiliations:** 1 Department of Clinical Pharmacy, Poona College of Pharmacy, Bharati Vidyapeeth University, Pune, Maharashtra, India; 2 Indian Institute of Science, DST Center for Policy Research, Bengaluru, India; 3 Institute of Public Health, Bengaluru, India; Qatar University College of Medicine, QATAR

## Abstract

India has a massive burden of rare diseases (RDs), with an estimated 96 million people living with RDs but limited options for treatment. Repurposing drugs used for other common conditions are essential alternative for RDs due to their cost-effectiveness and reduced timeline. India’s patent regime prevents the evergreening of drugs, and a large generic manufacturing industry provides ample opportunity to explore the potential of repurposed drugs for treating RDs, known as repurposed orphan drugs (ROD). However, there is no portal or other source for information on orphan drugs in India. This study assesses the availability of RODs in India through quantitative analysis. In the absence of a separate orphan drug designation in India, we consider USFDA-approved orphan-designated products as the reference. We searched USFDA-approved RODs in recognized sources in India, such as CDSCO, AYUSH gazette, FSSAI, and Indian Pharmacopeia, which provides a list of drugs approved for marketing in India. We classified the drugs into separate groups based on their record from different sources and explored the regulatory implications of the differential representations. We found that almost 76% of the USFDA-approved RODs are listed in one of the Indian regulatory bodies’ records either in the same form (61%) or in a different fixed-dose combination. For 67 drugs no information was found, these drugs have to be imported for use. Only 17 of the 43 RDs mentioned in the National Policy for Rare Diseases, matched the indications listed for approval of one of the 279 RODs identified in the study. This underscores the scarcity of RD treatment and the pressing need for accessibility in India. This information on RODs will help relevant stakeholders to efficiently manage RDs in India. The study also highlights existing gaps in Indian regulatory databases that limits access to accurate information about the availability of drugs.

## Introduction

Rare diseases (RD) are severe life-debilitating conditions that infrequently occur in a population. They are individually uncommon but cumulatively affect a substantial population [1]. Drugs for treating RDs are known as ’Orphan Drugs’ (ODs) because traditionally, due to small patient-size and resultant poor economic viability, pharmaceutical companies and the government had limited research and development interest in these drugs. India has a massive burden of Rare diseases (RD), with about 96 million people living with RD [2]. Despite such a huge burden, until recently, there has not been much attention from the government and pharmaceutical industry to address the needs of RD patients in India. In 2019, the Central Drugs Standard Control Organisation (CDSCO), the drug regulatory body of India, included special provisions for orphan drug approval and defined ODs in India as "drugs that are intended to treat a condition that affects no more than 0.5 million people" [3]. In 2021, the National Policy for Rare Disease (NPRD) was released. The lack of access to ODs for RDs in the country is acknowledged within the NPRD stating, "drugs for the treatment of RDs are exorbitantly costly and not universally available & accessible" [2]. NPRD further acknowledges that there are no domestic manufacturers for ODs in India except for Food for Special Medical Purposes (FSMP) for small molecule inborn errors of metabolism [2].

The lack of treatment for RDs is a global challenge, as 95% of RDs do not have an approved treatment [4]. Of the approved drugs, novel ODs are prohibitively expensive and range to hundreds of thousands of dollars. For example, Luxturna, a gene therapy to treat inherited retinal disease, costs around $850,000 per one-time treatment [5]. Such cost of treatment makes most of the novel therapeutics for RDs inaccessible in low and middle-income economies. Novel orphan drug development is more complex than other large-market drugs. The major challenges are a small, geographically dispersed patient population, poorly developed study endpoints, insufficient patient data, and inappropriate control groups. Repurposing drugs used for other common conditions has been considered an essential alternative for RDs due to their cost-effectiveness and reduced timeline resulting in higher success rates than novel drugs [6,7]. The development of systematic approaches to repurposing compounds has led to the identification of promising candidate drugs with the potential to treat RDs [6,8]. The US Food and Drug Administration (USFDA) has had several legislative initiatives, such as the 505 (b) (2) application, the Dormant Therapies Act (2014/2015), and the Orphan Product Extensions Now Accelerating Cures & Treatments Open Act (2014/2017) to support the repurposing of drugs.

India has a vast potential to use repurposed orphan drugs (RODs) as it is the biggest global manufacturer of generic medicines [8]. Many RODs out of the exclusivity period are possibly manufactured and available in India and used for other conditions. Further, the patent regime in India prevents the evergreening of pharmaceutical products’ patents that allows the protection of incremental changes in previously approved drugs [9]. This will enable the generic manufacture of many RODs in India. There is also a provision for ‘Subsequent new drug application’ for approval of an already approved new drug (within 4 years), with new claims, namely, indications, dosage, dosage form, and route of administration [10]. NPRD also supports research for repurposing drugs [2]. Further, the Department of Science and Technology, Government of India, has recently announced a call to support milestone-driven proposals for the listed rare diseases to develop off-patent generic drugs [11].

However, there is an absence of a dedicated orphan drug approval system or information portal in India. As such, information on the availability of these drugs is difficult to access [12]. There has been no study to assess the availability of ODs in India. Studies on the availability of ODs conducted in countries with new or upcoming RD and OD policies such as China and Turkey have helped to highlight the need and gaps in the ecosystem for better policy implementation [13,14]. Information on the availability of ODs is essential for clinicians, pharmacists, researchers, and patients to enable easy access to managing these diseases. This information will also guide the industry and researchers to identify drug candidates for which generics can be launched in India. Therefore, we have conducted this quantitative study to assess the availability of repurposed ODs in India. We searched the FDA-approved ODs in publicly available databases of Indian regulatory bodies. Listing of these drugs in one of the public databases is an important factor in determining their presence in India. We then classified the drugs into separate groups based on their record in different sources. Further, for the groups that did not show a straightforward match in the Drugs@CDSCO list, we explored the possible regulatory pathways that would help to make the drugs available in the Indian market. Thus overall, the study’s primary goal is to discover all FDA-approved RODs that are presently accessible in India and investigate the regulatory authorities involved in their approval. Furthermore, the study aims to extensively explore the necessary regulatory pathways that would facilitate the availability of these drugs in the Indian market.

## Materials and methods

**Fig 1:**
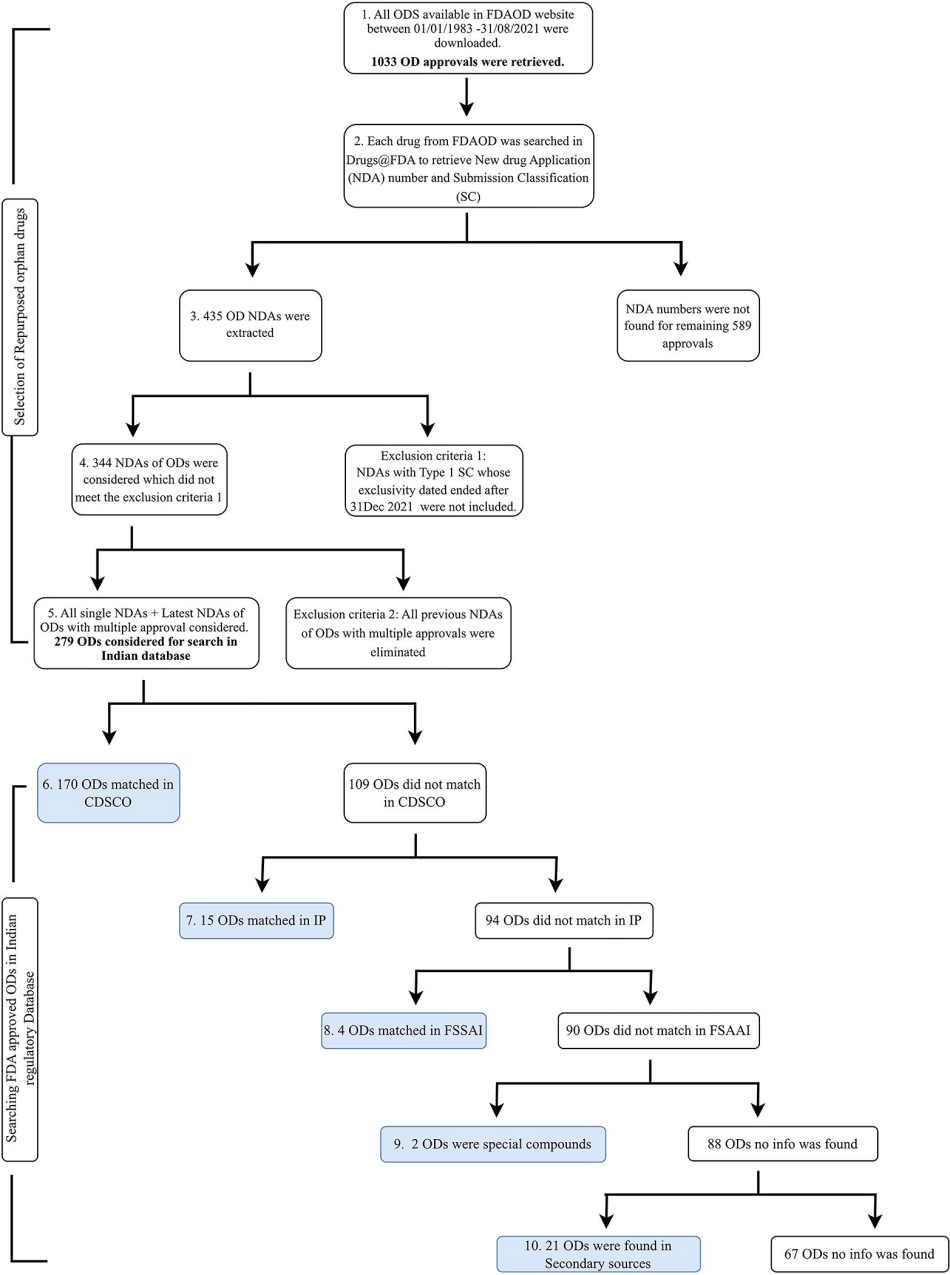


This is a quantitative cross-sectional study from the perspective of data collection. The information from The United States Food and Drug Administration (USFDA) was collected within a single time frame and associated with the data on the drugs available in India. No human participants were involved. We ought to identify how many repurposed orphan drugs are available in India. However, unlike the USFDA, the Indian regulatory body for pharmaceuticals, CDSCO does not provide any special status such as an ‘orphan designation’ to a drug or biological product to treat an RD. Therefore, we chose to use USFDA-approved orphan drugs as a reference to identify repurposed orphan drugs available in India. Thus, to identify repurposed orphan drugs that are available for marketing in India, we intended to select (i) all small molecule drugs that had received approval under orphan designation by the USFDA,(ii) all drugs in (i) with submission classification (SC) of Type 2 to Type 7 and (iii) drugs in (i) with Type 1 SCs whose exclusivity ended before 31^st^ August 2021. Submission classification details and their relevance in deciding the inclusion criteria of the study are given in S1 Data. Description of the method is given in Fig 1 and the steps are described below.

1. A list of all orphan drugs was downloaded from the FDA’s Orphan Drug Designations and Approvals database (FDAOD) website [15]. The start date was set to default to 01/01/1983 to the end date of 31/08/2021. The spreadsheet was downloaded on 01/09/2021. The list downloaded from the FDAOD consisted of 1033 approvals for orphan-designated drugs.
2. The products listed in the downloaded spreadsheet from FDAOD were searched on the Drugs@FDA site [16]. The trade name was initially used for the search because the NDA was not available in the FDAOD list. The first iteration was done using Python version 3.09.1 in Visual Studio code, which extracted the NDA numbers for most products and was followed by intensive manual intervention. Some of the trade names listed in the FDAOD list were not found on the Drugs@FDA site. It is possible that these products belong to categories that are not included in the Drugs@FDA site such as blood and blood products intended for infusion, biologics licensed application, plasma derivatives, vaccines, and allergenic products (for example, allergen extracts for diagnosis and treatment), cellular and gene therapy products. Subsequently, for each trade name, the marketing approval dates listed in the FDAOD were matched with the NDA Action Date in the Drugs@FDA site to identify the correct NDA number that has received the orphan approval. The corresponding submission classification listed in the Drugs@FDA was also extracted.
3. Comparing the list with Drugs@FDA extracted NDAs for 435 drugs. This eliminated the products mentioned above that are not listed in Drugs@FDA.
4. Additionally, all Type 1 drugs whose exclusivity ended after 31st August 2021 were eliminated leading to 344 drugs.
5. The next round of elimination was done to select the latest approval NDA in cases where multiple approvals were received for an orphan indication. For example, Mesna had type 1 (Approval date: 12/30/1988) and type 3 (Approval date: 03/21/2002) approvals but the latest approval was considered i.e. its type 3 approval. This was to confirm that the drug was out of the marketing exclusivity period for its latest FDA approval. This helped to remove redundancies in the list caused by drugs with multiple NDAs, and a final list of 279 drugs was obtained.
6. This list of 279 ODs was used as a reference to search for repurposed orphan drugs recorded in different regulatory sources in India as listed in Table 1. Each drug from the list of 279 ODs was first searched in CDSCO. CDSCO is the main drug and clinical trials regulatory body of India [17]. Hence primarily Drugs@CDSCO portal was considered to match the 279 approvals.

For those drugs that did not have any match in CDSCO, alternative sources were compared like the Indian Pharmacopoeia (IP) [18], the Food Safety and Standards Regulations, 2016 [19], and the Ayurveda, Yoga and Naturopathy, Unani, Siddha and Homeopathy (AYUSH) ministry website [20].

7. After CDSCO, the IP was the next source of choice as it is maintained and updated regularly by the Indian Pharmacopoeia Commission (IPC) autonomous body under the Government of India to set standards for drugs included in Schedule II of the Drugs and Cosmetics Act, 1940. IPC publishes official documents for improving the quality of medicines by adding new and existing monographs in the form of IP. In this study, we referred to the Eighth Edition of IP published by the IPC, Ghaziabad, in 2018.
8. The next source we searched was the Food Safety and Standards Regulations, 2016 (FSSAI Regulations, 2016) published by the Food Safety and Standards Authority of India (FSSAI). FSSAI overviews science-based standards for articles of food and regulates their manufacture, storage, distribution, sale, and import to ensure the availability of safe and wholesome food for human consumption. It includes regulation of health supplements, nutraceuticals, food for special dietary use, food for special medical purposes, functional food, and novel food.
9. Next, the medicines used in Ayurveda, Yoga, Naturopathy, Unani, Siddha, and Homoeopathy were also analysed by information from the Ministry of AYUSH website. These are the alternative systems of medicine prevalent especially in the South Asian countries on the global map [21]. Hence, these too were attempted to match with the FDA OD list.
10. For those drugs with no matches in the above sources, secondary sources were also considered, such as the pharmaceutical manufacturing or marketing company’s websites and reputed newspaper articles. Those that did not match any of the categories were considered to be not unavailable in India and could be procured only through direct import.

**Table 1:** List of the sources that were searched to capture a listing of USFDA-approved repurposed orphan drugs in India, with a description of datasets and their relevance to the study.

Source Name	Source Description	Data Description	Relevance of the source in this study
Central Drugs Standard Control Organisation https://cdscoonline.gov.in/CO/Drugs	CDSCO is India’s national regulatory body for cosmetics, pharmaceuticals and medical devices. Drugs@CDSCO provides the list of approved drugs in India.	Drugs and Fixed dose combinations (FDCs) approved for marketing in India	All FDA-approved ODs that are listed in Drugs@CDSCO are expected to be available for marketing in India as it is the primary official regulatory database. So, it was our primary choice of search.
Indian Pharmacopoeia (IP) https://www.ipc.gov.in	IP is developed by the Indian Pharmacopoeia Commission (IPC), which is an Autonomous Institution of the Ministry of Health and Family Welfare, Govt. of India. IPC was created to set standards for drugs in the country. Its basic function is to regularly update the standards of drugs commonly required for the treatment of diseases prevailing in this region.	IPC publishes official documents for improving the quality of medicines by adding new and existing monographs in the form of Indian Pharmacopoeia (IP).	IP is a regularly updated public database therefore we next searched IP for those FDA-approved OD that were not available in Drugs@CDSCO
Food Safety and Standards Authority of India https://www.fssai.gov.in/cms/food-safety-and-standards-regulations.php	Responsibility for ensuring compliance with the FSS Act, 2006 rules and regulations made thereunder by the FBOs	Nutraceuticals approved for marketing in India	Special foods and nutraceuticals are not listed in CDSCO and IP so FSSAI was searched
Secondary Sources: Websites of the companies and newspaper articles	These include information from sources such as reputed newspapers and public domain of the companies’ websites.	Drugs listed as available in India but are neither mentioned in Drugs@CDSCO, IP or as Nutraceuticals	Some of the drugs are available through compassionate use programs even if they do not yet have a marketing approval from one of the regulators
Other sources (Special cases) https://www.usp.org/sites/default/files/usp/document/harmonization/excipients/pf305-dehydrated-alcohol.pdf https://legislative.gov.in/sites/default/files/A1985-61.pdf	These include: 1) Dehydrated Alcohol: As defined in the US Pharmacopeia (The US Pharmacopeia is used to set global drug standards) 2) Cannabidiol is regulated by the Government of India under the Narcotics Drugs and Psychotropic Substances Act, 1985		

Following the search for the 279 drugs in the Indian database, we attempted to search the patent status of these drugs in India. The Indian “composition of matter” patent information (COM) corresponding to these 279 drugs was collated. It was first checked in PAT-INFORMED, a database that provides information on the World Intellectual Property Organization’s (WIPO) own global database, by entering a medicine’s INN (International Non-Proprietary Name) to obtain its patent in India. For those drugs whose information was not found in this step, sophisticated databases like Derwent Innovation and Patsnap were explored. Lastly, Orange book archives and the current version of Orange Book were checked to get US COM Patents corresponding to each drug. Then, the International Patent Documentation was checked to locate Indian patents. For the rest of the drugs, secondary patents were found (covering formulation, and preparation techniques) instead of COM patents. The patent search exercise was outsourced to GreyB a consulting firm with deep expertise in extracting patent information.

## Results

**Fig 2:**
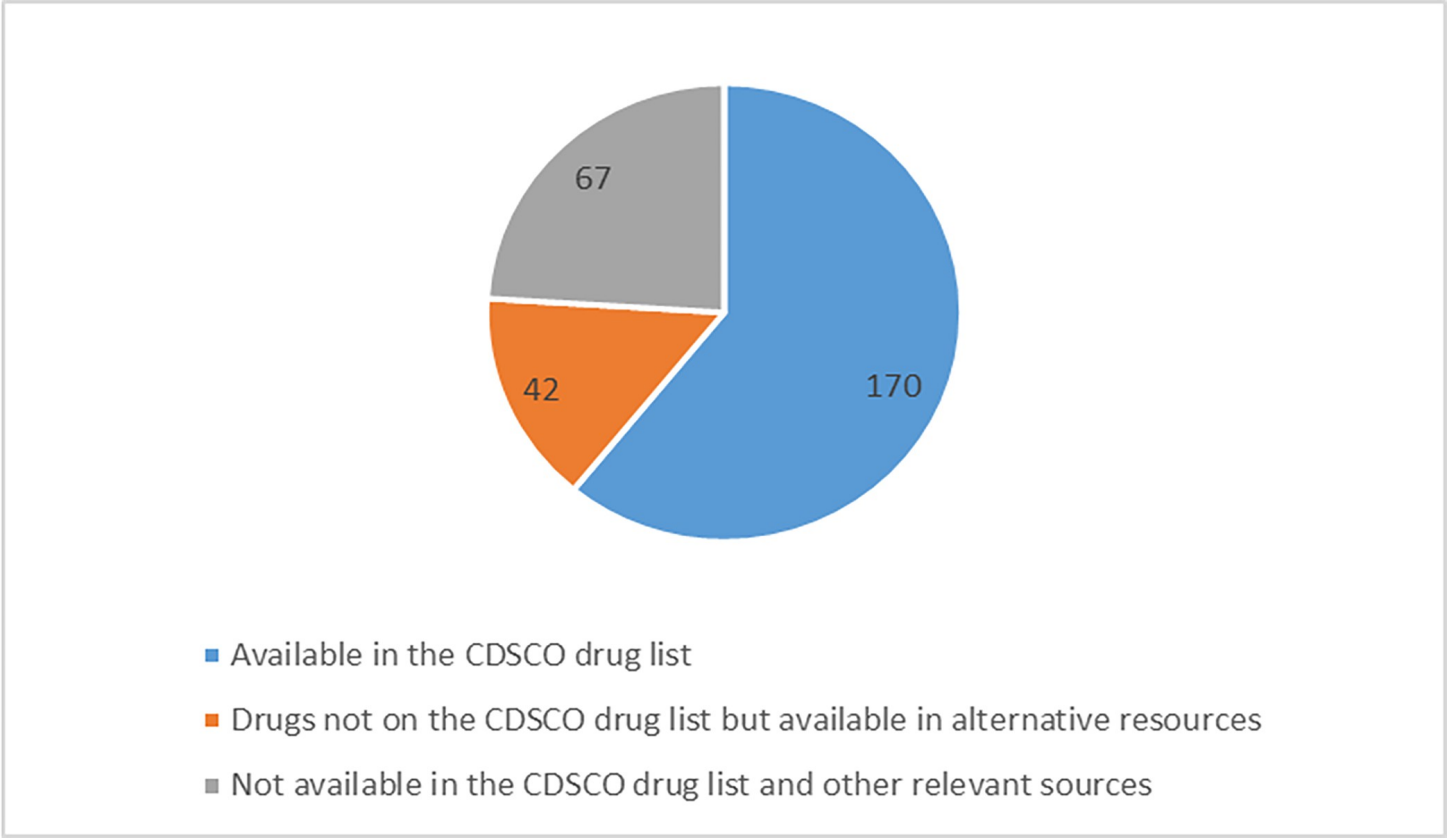
Presence of 279 orphan drugs in CDSCO and other sources.

**Fig 3:**
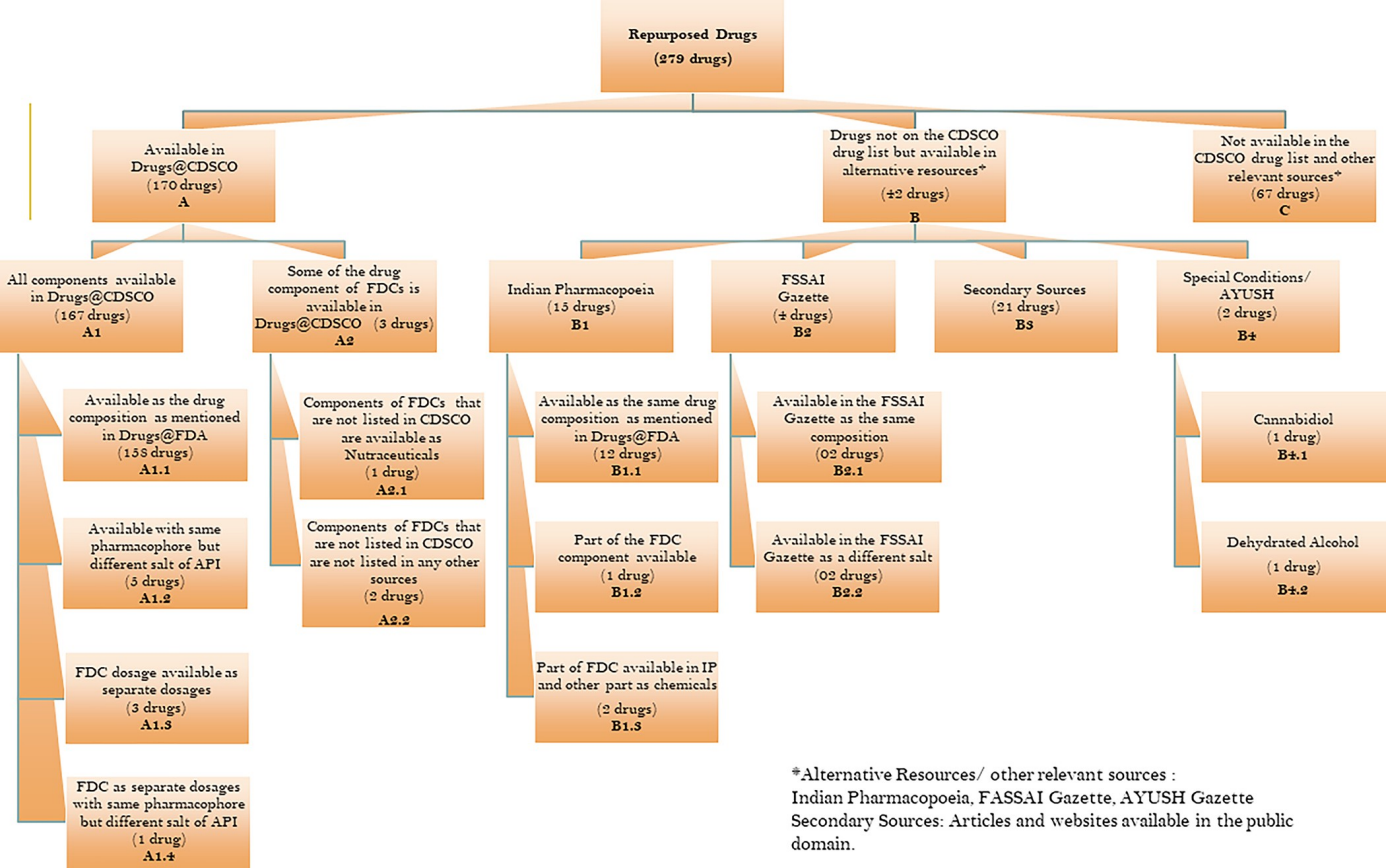
Categorization of 279 repurposed orphan drugs into groups based on their availability in different sources in India.

We found that out of 279 products mentioned above, 170 were available in the Drugs@CDSCO list. The remaining 109 products were searched in different sources, resulting in a match of another 42 products. However, 67 products were not listed in any searched sources and are considered unavailable in India but can be procured only through direct import. Fig 2 shows the percentage distribution of 279 drugs in CDSCO and other sources. All the products were identified in different categories based on their match to different sources described in Table 1. We broadly describe each of these categories in Fig 3, and Supplement 2 in S2 Data which indicates their presence/absence in the Indian Market.

### Group A: Available in the Drugs@CDSCO list

170 products matched either partially or completely with the CDSCO list, these were categorized as Group A. For 167 products the entire drug or all the components were found listed in CDSCO and were categorized as group A1. For the remaining three products that are fixed-dose combinations (FDCs), only some of the components were listed in the CDSCO list and were categorized as A2. We classified group A1, into further 4 groups which we describe here:

A1.1: 158 products were mentioned with the same salt composition as mentioned in Drugs@FDA.

A1.2: For five products, the pharmacophores were the same, but the salt composition was different. These included *Calcitonin—human for injection*, *hydroxyprogesterone caproate*, *Ibuprofen lysine*, *Caffeine*, and *paricalcitol* available in the Drugs@FDA list. However, in the CDSCO list, the following Active Pharmaceutical Ingredients (APIs) were available respectively: *Salmon calcitonin injection*, *hydroxyprogesterone*, *Ibuprofen*, *Caffeine citrate*, and *calcitriol*.

A1.3: In cases of another three FDCs, the products were available as separate dosage forms rather than a single dosage formulation. These included *Trametinib and Dabrafenib*, *Ledipasvir and Sofosbuvir* and *magnesium chloride*, *sodium bicarbonate*, *sodium chloride (Bicarbonate infusion*).

A1.4: In the case of *Cytarabine and daunorubicin citrate*, both drugs were available in separate dosage forms but *daunorubicin citrate* was available as *daunorubicin* in the CDSCO list.

In category A2, only some of the components from FDCs were either not available or not designated as a drug by CDSCO. The three cases were

- (A2.1) The FDC mentioned in the FDA OD list is *Citric acid*, *glucono-delta-lactone and magnesium carbonate*. However, in the CDSCO list, only *Citric acid and magnesium carbonate* were available but *glucono-delta-lactone* is available in the approved list of nutraceuticals in the FSSAI Regulations, 2016.

(A2.2) The FDC mentioned in the FDA OD list are S*ofosbuvir and velpatasvir*, Sofosbuvir is available in the drugs@CDSCO list while Velpatasivir is not listed. In the second case, the FDCs mentioned in the FDA OD list are *Colfosceril palmitate*, *cetyl alcohol and tyloxapol*. However, tyloxapol is neither on the approval list of CDSCO nor any other alternative sources.

### Group B: Drugs not on the Drugs@CDSCO list but available in alternative resources

109 products were not listed on the Drugs@CDSCO list. Hence, they were searched in relevant sources such as IP, FSSAI Regulations, 2016, AYUSH-approved drug lists, the public domain of pharmaceutical company websites, and reputed newspaper articles. These drugs have been classified into 4 groups based on the source where 42 products were found to be listed:

B1: IP was the first alternative source that was referred to after CDSCO because it is the only publicly listed source other than CDSCO. We found a match for 15 drugs in this source for either all components or some of the components of the drugs. Based on the match the subcategories identified are described below:

B1.1: 12 products from the FDA OD list were available as the same drug composition in the IP list.

B1.2: FDC combination mentioned in the FDA OD list is *Sodium nitrite and sodium thiosulfate*, however in IP only *sodium thiosulfate* was available.

B1.3: In the case of 2 FDCs, some components of the FDC are listed in IP whereas other components are widely available as chemicals in the country. FDC combinations mentioned in the FDA OD list are *benzoate/phenylacetate (Sodium salt);* and *sodium sulfate*, *potassium sulfate*, *and magnesium sulfate*. The Sodium salt of Benzoate is mentioned in the IP but *Sodium phenylacetate* is not mentioned. Similarly, Magnesium sulfate is mentioned in the IP whereas *Sodium Sulfate* and *Potassium Sulfate* are not mentioned. However, In India, *Sodium phenylacetate*, *Sodium Sulfate* and *Potassium Sulfate* are widely sold as bulk chemicals in the country [22].

B2: The third source that was referred to find the FDA OD listed products were FSSAI Regulations, 2016. Nutraceuticals are products that have been approved by FSSAI to provide a physiological benefit and help maintain good health.[23] Four products in the FDA OD list were found to be listed in the FSSAI Regulations, 2016. Among them, two products *zinc acetate* and *L-glutamine*, were available in the FSSAI Regulations in the same composition as they were listed in FDA OD. Whereas the other two drugs *Glutamine* and *Betaine were available as a different salt of L-glutamine* and *Betaine HCl* respectively in the FSSAI regulations.

B3: 21 drugs were not listed in any of the above-referred sources but were found to be listed on their respective pharmaceutical company’s websites and other publicly available sources of information, such as reputed newspaper articles listed in Supplement 2 in S2 Data. Information about these drugs indicated the possibility of their presence in the Indian Market.

### Group C: Not available in the CDSCO drug list and other relevant sources

67 out of 279 drugs are neither available in the CDSCO drug list nor in any other sources, which indicates that they are not approved for marketing or manufacture in India. These drugs have to be imported directly only. Some of the drugs were listed in import-export portals, global pharmaceutical distributor’s websites, and other B2B platforms as cited in S1 Data. However, we could not further verify their presence in the Indian market and hence marked them as not easily accessible to the patients.

### Primary patent search

A patent search for 279 drugs in Indian databases retrieved data for only 20 drugs. For the remaining 259 drugs, a “composition of matter” patent information was not found. The data correlated with 3 (A1.1, B3, and C) of the categories of drugs classified in Table 2.

**Table 2:** Indian composition of matter patent details of 20 drugs for which data was found in the Indian database.

Category from Fig 2	Description of the category	Drugs in each category	Drugs with a patent expiration date before 1^st^ Dec 2022 (Available for generic manufacture)	Drugs with a patent expiration date after 1^st^ Dec 2022 (NA for generic manufacture)	Drugs for which patent expiry dates were not found in the Indian database
A1.1	All components available in the CDSCO list	14	03/14	06/14	05/14
B3	Secondary Sources	02	00/02	01/02	01/02
C	Not available in the CDSCO drug list and other relevant sources*	04	02/04	01/04	01/04

14 drugs were from the A1.1 category, meaning all the drugs’ components were listed in CDSCO. Two drugs were listed in secondary sources (B3 category), and four drugs were not found in any relevant sources. Among the 14 drugs, for three drugs, Glatiramer Acetate, Crizotinib, and Lenalidomide, the patent expiration date was before 1^st^ Dec 2022 hence these drugs should be available for manufacturing as generics in the country unless they are protected by any other secondary patent. For another six of the 14 drugs, the patent expiry date was beyond 1^st^ Dec 2022. For the remaining five drugs and two drugs in Category B and C, information about patent expiry dates were not found. For these seven drugs, “composition of matter” patents are still in the application stage (not granted yet).

## Discussion

Identifying FDA-approved repurposed orphan drugs is important in the Indian context as the off-label use of drugs is illegal in India and Indian law does not currently allow drugs to be prescribed for indications for which they have not been approved [24]. However, the New Drug and Clinical Trial Rule (NDCTR 2019) allows the marketing of any drug in India that has received approval for an orphan indication in certain other countries. Thus USFDA-approved repurposed orphan drugs can be easily made available in India. Thus, it is important to bring attention to the availability status of FDA-approved drug repurposing and off-label uses for RDs where sometimes it may be the only available treatment.

We identified 279 (27%) small molecule drugs out of 1033 orphan-designated drugs approved by the USFDA till 2021, as repurposed for orphan use, and are out of marketing exclusivity period by 2020. This shows a slight rise from the data reported by a study in 2017, where repurposed orphan drugs accounted for 20% of all orphan drugs [25,26].

We found that almost 76% (212) of the identified repurposed drugs are listed in one of the referred Indian regulatory bodies registries either in the same form or in a different FDC. Of the 279 drugs, 61% (170) are approved by CDSCO and are available on the CDSCO website (cdscoonline.gov.in/CDSCO/Drugs.) Being listed in the CDSCO list means the drug has been registered for marketing in India. However, it does not ensure their easy availability in the Indian market which is often regulated by various factors. A few approved products listed in Indian databases were not available in the market [27]. For example, Miltefosine is used to treat leishmaniasis in India, but due to the presence of alternative therapies and a small margin on the drug, its availability is limited [28].

Nevertheless, being listed in the regulatory databases ensures that these drugs have the regulatory approval for being manufactured and marketed in India. If awareness about their orphan use is communicated to patient organizations, clinicians, and the pharmaceutical industry, they can help to drive the availability of these medicines in the Indian market. For example in the early 1990s Sandoz’s Intravenous Immunoglobulin, was very expensive and had to be imported. However, continuous efforts by the patient groups led to its manufacture in the country and made it more affordable [29].

For 60% (category A1, 167 out of 279) either the FDC was listed in the CDSCO list or all the components were listed. A1.1 group compromised 158 drugs that showed a straightforward match with the CDSCO list where similar compositions were listed in the CDSCO list. Thus this list comprises 57% of the total drugs that ideally should be easily available in India.

However, for the remaining 12 drugs in category A, some of the components of FDCs were either not listed in CDSCO or listed, but the FDC was not listed. The requirements for permission to manufacture/ import and market any FDC in the country are described by CDSCO in the Policy Guidelines for Approval of Fixed Dose Combinations (FDCs) in India (CDSCO FDC Policy) [30]. It has classified FDCs into 4 broad categories and described the required data/ evidence for marketing approval/permission to conduct clinical trial/BA-BE studies under each category or sub-category.

Thus, in the case of 10 drugs belonging to the categories A1.2, A1.3, A1.4 and A2.1, all APIs are approved/ marketed in India individually, but the FDC is not approved for marketing. However as these FDCs are approved by USFDA and marketed with established safety and efficacy in humans, marketing approvals for such drugs can be sought after the submission of requisite documents as described under Category II A.

For the remaining two FDCs in A2.2, (i) sofosbuvir/velpatasvir and (ii) Colfosceril palmitate, cetyl alcohol, and tyloxapol, one of the APIs are not available in the Drugs@CDSCO list. However, the FDC, sofosbuvir/velpatasvir, is listed in the published list of Fixed Dose Combinations Approved By DCG (I) Since 1961 to 28th June 2019 released [31]. Sofosbuvir and Velpatasvir is a well-known FDC combination and is widely used for treating viral Hepatitis C infection in India. Colfosceril palmitate, cetyl alcohol, tyloxapol, is classified under category I of CDSCO FDC Policy, where one or more APIs of the FDC is a new drug (as per Rule 122E of D&C Rules, 1945) not approved in India. For such FDCs to be approved for manufacture/ import and marketing, the data required to be submitted will be the same as that for a new chemical entity (NCE) as per Schedule Y.

For the remaining 109 drugs alternative sources were searched, and 15 drugs were found listed in the IP. Other than drugs and FDCs approved by CDSCO, IP also lists drugs used in national health programs of India, drugs listed in the essential medicines list and drugs considered appropriate by the IPC [18]. As to why these drugs were not listed in Drugs@CDSCO is not very clear and merits further investigation. Nevertheless, being listed in IP suggests that these drugs would be present in the Indian market.

Another 4 drugs were found to be approved as Nutraceutical in India. FSSAI defines Nutraceuticals as “naturally occurring ingredients that are extracted, isolated and purified from food and non-food sources and consumption in measured amounts provide physiological benefit and helps maintain good health” [19]. However, in USFDA, these drugs were not identified as ‘nutraceuticals’ or any similar classification. USFDA does not classify any product as ‘nutraceuticals’ rather extracts, concentrates, or combinations of vitamins, minerals, botanicals, herbs, or dietary substances for use by man to supplement the diet by increasing the total dietary intake are identified as ‘dietary supplements’[32]. Additionally, the FDA also identifies products that include plant materials, algae, macroscopic fungi, and combinations as ‘botanical drugs’ [33]. Nevertheless, nutraceuticals are known to provide enormous benefits, especially in RD management where curative treatment might not be available [34]. Many RD patients in India have acknowledged the benefits of such medicines in improving their quality of life [26]. India has a huge potential to explore the benefits of traditional medicine systems like Ayurveda, Unani, Siddha etc. AYUSH ministry can play a major role in setting up a parallel standard of care for the treatment and management of RDs using these alternative systems of medicine. In fact, in 2019, the government agreed to a Madras High Court’s plea to constitute a committee of experts in Ayurveda, homeopathy and other alternative medicines (AYUSH) to ascertain available remedies for people affected with lysosomal storage disorders [35].

21 drugs classified under the B3 category were not found in any of the primary sources that we searched viz. CDSCO, IP, FSSAI. However, these drugs were reported to be approved by CDSCO in other secondary sources as indicated in S2 Data. The reason for these drugs not being listed in the primary sources is unclear nevertheless, delay in the updation of the primary sources could be one potential cause.

Cannabidiol is regulated by the Central Bureau of Narcotics under the Narcotic Drugs And Psychotropic Substances, Act, of 1985. It is therefore not listed in the CDSCO drug list however historically, India has continued to produce and use Cannabis for medicinal and nutritional purpose and various indigenous systems of medicine such as Ayurveda, Unani, Siddha also documents the use of cannabis in treating many disorders [36]. Thus Cannabidiol should be easily available to patients in India.

Thus 172 drugs(A1.1, B1 and B2) were listed in one of the Indian regulatory databases in the same form as approved by the USFDA. These drugs are therefore expected to be easily available in the Indian Market depending on their patent status. However, the Indian composition of matter information was found for only 20 drugs. Among these only three drugs were found to be listed in CDSCO with expired dates of ICOM patent. So we assumed these three products should be easily available on the market. These three products are: Glatiramer Acetate, an immunomodulator drug that is used to treat multiple sclerosis; Lenalidomide used to treat multiple myeloma, smoldering myeloma, and myelodysplastic syndromes and Crizotinib used for treating anaplastic lymphoma kinase. The primary reason behind not finding COM Indian patents for the rest 259 drugs is because Indian patent laws did not allow any product patents till 2005 [37]. Hence, no Indian family member has been filed before 2005. However, the absence of ICOM patent doesn’t assure that these drugs are open for generic manufacture as some of the drugs might have some secondary patents. For example, if the ICOM Patent for a hypothetical X drug either expired or did not exist, but there is a secondary Indian patent, which protects tablet formulation of X drug. In that case, generics companies have 3 options– 1) they can wait till the expiry of this secondary patent, 2) they can license the patent owner, and 3) they can invalidate the patent before launching the tablet version of X drug. However, they are allowed to launch any other formulation (e.g. capsule, syrup etc.) in the Indian market [37].

Finally, 67 drugs were not listed in CDSCO or any other sources, these drugs have to be imported for use. In the case of RDs, many drugs that are not permitted to be imported or marketed in the country are required exclusively for the treatment of patients to save their lives. Permit for import of such drugs in small quantities for personal use is facilitated by the Drugs and Cosmetics Rules, 1945 through Form 12B, which can be obtained from the office of the Drugs Controller General (India) or designated Port Offices of CDSCO [38]. Among these five drugs lomitapide; glycerol phenylbutyrate; tiopronin; calcium, magnesium, potassium, and sodium oxybates; cobicistat were mentioned in a CDSCO letter about having approval for manufacturing and packing in India [39–42] for export to EU, but they were not listed in CDSCO drug list.

Description of the categories and corresponding suggested regulatory interventions are listed in Table 3.

**Table 3:** Description of the categories defined in the study to assess possibility of market accessibility in India.

Category	No of Drugs	Description of category	Possibility of market availability	Regulatory interventions required
A1.1	158	Listed in CDSCO as the same dug composition as in Drugs@FDA	High	Ideally should be easily available for marketing
A1.2—A1.4	9	All components listed in the CDSCO list but not in the same FDC form	Medium	Marketing approvals can be sought after the submission of requisite documents as described under Category II A of CDSCO FDC approval policy guidelines
A2.1	1	One of the FDC component is listed as a nutraceutical	Medium	
A2.2	2	Some of the FDC component are not listed in any Indian source	Low	Based on category 1 of CDSCO FDC Policy, data required to be submitted for marketing approval is the same as that for a new chemical entity (NCE) as per Schedule Y
B1.1	12	Available in IP as the same drug composition as in Drugs@FDA	High	Ideally should be easily available for marketing
B1.2-B1.3	3	Part of the component of the FDC listed in the IP	Low	Based on category 1 of CDSCO FDC Policy, data required to be submitted for marketing approval is the same as that for a new chemical entity (NCE) as per Schedule Y
B2.1	2	Available in Neutraceutical Gazette as the same composition	High	FSSAI and AYUSH should undertake an evidence-based approach to provide management products for RDs that enhance the QOL of patients
B2.2	2	Available in Neutraceutical Gazette as a different salt	Medium	
B3	21	listed in Secondary sources	medium	Availability status unknown
B4	2	Special condition (canabidiol, dehydrated Alcohol)	Available in the market	Regulated by the Central Bureau of Narcotics under the Narcotic Drugs And Psychotropic Substances, Act, of 1985
C	67	Not listed in any of the referred Indian sources	Low	Permit for import of such drugs in small quantities for personal use is facilitated by Drugs and Cosmetics Rules, 1945 through Form 12B, which can be obtained from the office of the Drugs Controller General (India) or designated Port Offices of CDSCO

As mentioned above, waiver of clinical trials of FDA-approved ODs in NDCTR 2019 regulations should enable easy import of ODs that do not have CDSCO approval yet [24].

NPRD mentioned 43 conditions in the policy document as RDs in India, 17 of them had one or more drugs in the cohort of 279 RODs considered in this study (S3 Data). Some of these drugs were proposed for use as adjunctive therapy such as sodium phenyl butarate, Nitisinone; whereas some were the only available treatment options and some such as tolvaptan significantly reduced disease progression. Thus, availability and accessibility to these drugs is critical for patient care. Of these, only nine drugs were classified in the high probability groups (A1.1, B1, B2) which ensure that these drugs could be easily found in the Indian market. This highlights the huge gap existing in the availability of drugs for RDs in India. Therefore, urgent focus needs to be brought towards the development of new and repurposed ODs in India. To bring the focus of the pharmaceutical industry to ODs India needs a comprehensive orphan drug policy, as these policies have been highly successful in driving OD development in countries outside the US and Europe as well. Japan’s OD designation program was established in 1993 and in a span of 25 years 432 ODs were designated of which 322 (75%) were approved for marketing [43]. Brazil’s amazing achievement of approving 21 orphan drugs and 30 RD clinical trials in 2019 alone [44], can serve as an inspiration for India. Leveraging on one of the world’s biggest pharmaceutical manufacturing industries, India has the potential to take a lead in OD development in the global south.

Our study finds that 76% of USFDA-approved repurposed orphan drugs have approval for marketing from Indian regulatory bodies. It is crucial to make these drugs easily accessible to RD patients in India. Here in Box 1 we list the important recommendations from our observations in the study.

**Box 1: Recommendations**

Recommendations from the study:

 •
 • Similar to the National list of essential medicines, there needs to be a database of orphan drugs approved for the treatment of rare diseases which is regularly updated with the status of clinical trials, and domestic and international regulatory approval status.
 • The study found some discrepancies in the drug list between CDSCO and IP. Some drugs which are not listed in CDSCO are listed in IP and no possible explanation could be found. Such discrepancies should be looked into, and both databases should be updated. However, if there are valid reasons for the differential listings in both the databases the inclusion/exclusion criteria of the databases should be publicly made available.
 • AYUSH ministry should set up an evidence-informed parallel standard of care for the treatment and management of RDs using alternative systems of medicine, especially for diseases where no treatment is yet available.
 • Regulatory pathways of drugs from being approved by regulatory bodies such as CDSCO to being available to patient is not transparent and depends on multiple factors. The status of approval and market availability of the drugs should be made publicly available.
 • For 21 drugs secondary sources reported approval by CDSCO however these drugs were not listed in Drugs@CDSCO. Drugs@CDSCO should be made more robust to enable the updation of the new list in real-time.
 • The NPRD names only 43 rare diseases in the document. More than 8,000 RDs have been globally identified. There is an essential need for a functional Rare Disease registry that identifies and records all prevalent RDs in the country and maps corresponding drugs approved globally for each of them
 • Among the 43 rare diseases mentioned in NPRD, only 17 a drug could be matched in the cohort of 279 RODs. Therefore there is an urgent need to expedite orphan drug development in the country and identify commonly available drug candidates that can be repurposed for rare diseases.

## Conclusion

This study provides a comprehensive overview of the presence of USFDA-approved RODs in India. The classifications in this study help identify those approved for marketing and manufacture and can be made readily available to patients in India. We found that 76% of RODs approved by the USFDA have the requisite approvals from one of the regulatory agencies in India for either the entire composition or part of it. The study also highlights those products that are not approved but have the potential to address RDs based on experience in other countries. Regulatory authorities may prioritize the evaluation of these applications as some of these drugs might address diseases for which no alternative treatment is available. Further, various mechanisms provisioned by government Rules and Guidelines are discussed which can enable easy access to the drugs.

### Limitations

This study has some limitations in terms of the scope of the study:

1. Each country has its own definition of RDs and what may be rare in the USA might not be rare in India and vice versa. However, as India does not have a definition for RDs and the prevalence for most RDs is not available, we went on to consider all ODs designated by USFDA as the standard reference for this study.
2. We have not looked into the cost and pricing of the drug as it is beyond the scope of the study.
3. The data used may not be most updated as it is based on the latest data made publicly available by the concerned authorities. If there is a lag between the approval of drugs and making them available publicly on the website, such instances could not be covered.
4. Secondary patent information has not been retrieved so our discussion is limited to the primary patent of the drugs.

## Supporting information

S1 Data Description of submission classifications and their relevance to the inclusion criteria of the study. (XLSX)

S2 Data List of 279 repurposed orphan drugs from FDAOD and search for availability in the Indian sources. (XLSX)

S3 Data Rare diseases listed in the National Policy for rare diseases and their corresponding match in 279 repurposed orphan drug cohort of the study. (XLSX)

## Abbreviations

USFDA: The United States Food and Drug Administration
ROD: Repurposed Orphan drugs
RD: Rare Disease
CDSCO: Central Drugs Standard Control Organisation
NPRD: National Policy for Rare Disease
AYUSH: Ayurveda, Yoga and Naturopathy,Unani, Siddha and Homeopathy
FSSAI: Food Safety and Standards Authority of India
FDC: Fixed Dose Combinations
IPC: Indian Pharmacopoeia Commission
WIPO: World Intellectual Property Organization’s
INN: International Non-Proprietary Name
COM: Composition of matter
FSMP: Food for Special Medical Purposes
NDCTR: New Drug and Clinical Trial Rule
